# A Common Infection From an Unexpected Culprit: Metatarsal Osteomyelitis Caused by Streptococcus milleri in a Patient With Type 3c Diabetes

**DOI:** 10.7759/cureus.94233

**Published:** 2025-10-09

**Authors:** Austin P Runde, Sanchita Sen, Beatriz Maria Franco Hondermann, Camille Goodwyn, Agnes R Libot

**Affiliations:** 1 Department of Medicine, Loyola University Chicago Stritch School of Medicine, Maywood, USA; 2 Department of Medicine, Loyola University Medical Center, Maywood, USA

**Keywords:** bacterial infections, diabetic foot infection, digital amputation, osteo-myelitis, pancreatogenic diabetes, streptococcus anginosus group, streptococcus milleri, type 3c diabetes mellitus

## Abstract

A male patient in his late 40s presented to the emergency department of an academic, tertiary medical center with a malodorous, full-thickness ulceration on the lateroplantar aspect of his right foot, exposing the fifth metatarsal head. He was also found to be in a hyperosmolar hyperglycemic state with a blood glucose level of 628 mg/dL (reference range: 70-100 mg/dL) secondary to uncontrolled pancreatogenic (type 3c) diabetes mellitus (T3cDM). Cultures from a bedside biopsy performed by our podiatry service grew *Streptococcus milleri* (*S. milleri*) and unidentified anaerobes. Despite early and broad-spectrum intravenous antibiotics, a partial fifth ray amputation with fillet toe flap was necessitated and performed without complication. With source control demonstrated by negative surgical margins, our patient was downgraded to a five-day course of oral amoxicillin-clavulanate and discharged two days after amputation. Our patient's case supports the notion that *S. milleri*, a commensal bacterium of the gastrointestinal (GI) and genitourinary (GU) tracts, may have an underappreciated ability to cause skin and musculoskeletal infections in the setting of severely elevated blood glucose levels.

## Introduction

Milleri-group (also called anginosus-group) streptococci (*Streptococcus milleri *(*S. milleri*)) are a subgroup of viridans-group streptococci. *S. milleri* includes the related bacteria *Streptococcus anginosus *(*S. anginosus*), *Streptococcus constellatus *(*S. constellatus*), and *Streptococcus intermedius* (*S. intermedius*) [1, 2]. *S. milleri* was first identified by Guthof in 1956 upon isolation from oral infections. These streptococci are unique in that they may be alpha-, beta-, or gamma-hemolytic and may express Lancefield antigens A, C, F, or G; they can also be Lancefield-nontypeable [3]. Since then, *S. milleri* has been identified as a commensal organism of the human gastrointestinal (GI) and genitourinary (GU) tracts, although not as part of normal skin flora.

While *S. milleri* is often implicated in deep-space, pyogenic infections, including those of the head, neck, abdomen, heart, and lungs, osteomyelitis caused by *S. milleri *is a rarely reported phenomenon [3-6]. Only a handful of *S. milleri* osteomyelitis cases have been found in the literature, some of which involved the vertebrae, skull base, femur, and hand phalanx [7-12]. To our knowledge, only one case of *S. milleri* osteomyelitis involving the metatarsal bones has been reported previously [13]. The most common cause of osteomyelitis is *Staphylococcus aureus *(*S. aureus*), but organisms such as coagulase-negative staphylococci, beta-hemolytic streptococci, enterococci, aerobic gram-negative bacilli (such as *Enterobacter *spp.), and anaerobic gram-negative bacilli (such as *Peptostreptococcus *spp. and *Bacteroides *spp.) are also commonly isolated. While osteomyelitis arising from hematogenous spread is typically monomicrobial, osteomyelitis arising from contiguous spread, such as in the case of our patient, can be either poly- or mono-microbial [14, 15].

In diabetes mellitus (DM)-related foot infections (DFIs), which are typically polymicrobial and frequently progress to osteomyelitis, the most commonly isolated organisms include *S. aureus* (a constituent of normal skin flora), beta-hemolytic streptococci, *Enterococcus *spp., *Pseudomonas *spp., *Escherichia coli*, *Klebsiella *spp., and *Proteus *spp. [16]. While beta-hemolytic streptococci are frequently implicated in osteomyelitis and DFIs, the mixed-hemolytic* S. milleri* are very uncommonly reported in both infection types and may be more prevalent than previously believed [17].

Pancreatogenic (also called type 3c) DM (T3cDM) is an uncommon type of DM that occurs secondarily to severe dysfunction of the endocrine pancreas [18]. T3cDM is estimated to account for up to 10% of DM cases globally. While chronic pancreatitis is the most common cause of T3cDM, responsible for nearly 80% of cases, hemochromatosis, pancreatic cancer, cystic fibrosis, and iatrogenic surgical causes are also implicated. Since damage to the pancreas is the primary etiology of T3cDM, co-occurring insufficiency of the exocrine pancreas is frequently observed in T3cDM. Blood glucose levels in patients with T3cDM can be uniquely challenging to manage, given the reduced function of both alpha (glucagon+) and beta (insulin+) cells; hence why T3cDM is often known as "brittle diabetes". Moreover, T3cDM is often misdiagnosed as type 2 DM (T2DM) because T3cDM also typically occurs in adulthood, and there is overlap between the comorbidities commonly seen in patients with either type. Importantly, while insulin resistance may be present in T3cDM, it is not the primary etiology, as it is in T2DM [18].

## Case presentation

Patient description

Our patient was a male in his late 40s with a past medical history of chronic pancreatitis complicated by T3cDM and exocrine pancreatic insufficiency, below-knee amputation of the left extremity due to osteomyelitis of the left foot (performed three years prior), alcoholic hepatitis secondary to alcohol use disorder (~8.5 standard drinks (~119 g of alcohol)/day for two years)), painful peripheral neuropathy, *Clostridioides difficile* (*C. difficile*)-associated diarrhea, diverticulosis, hypertension, and hyperlipidemia. Home medications were atorvastatin 40 mg daily and pancrelipase 108,000 units with every meal. He reported previously using insulin and metformin (as well as glipizide and pioglitazone, which are generally avoided in T3cDM due to limited efficacy and side effects), but endorsed not having taken any of these medications for three weeks prior. His most recent HbA1c was 11.9% (reference for patients with DM: < 7.0%), measured 24 days prior. Chart review revealed a previous mention of "brittle DM" and a type I DM workup performed two years prior that was negative for islet cell, zinc transporter 8 (ZNT8), and islet antigen-2 (IA-2) autoantibodies, but positive for glutamate decarboxylase 65-kDa isoform (GAD65) autoantibodies at 0.03 nmol/L (reference: ≤ 0.02 nmol/L). Surgical history was significant for an exploratory laparotomy performed three years prior for intussusception (it was unclear whether the bowel was resected). Social history was negative for controlled substance use, positive for current tobacco use at 15 pack-years, and positive for alcohol use two weeks prior. Family history was significant for an unspecified heart failure in one biological parent and an unspecified lung cancer in the other biological parent.

Case history

Our patient presented to our institution's emergency department (ED) for treatment of an open, malodorous wound with an exposed fifth metatarsal noticed two days prior. He denied pain at the site, fever, chills, fatigue, and any inciting injury to the area. He endorsed a history of T3cDM but nonadherence to regular blood glucose checks and regular usage of antidiabetic medications.

Physical exam

**Figure 1:**
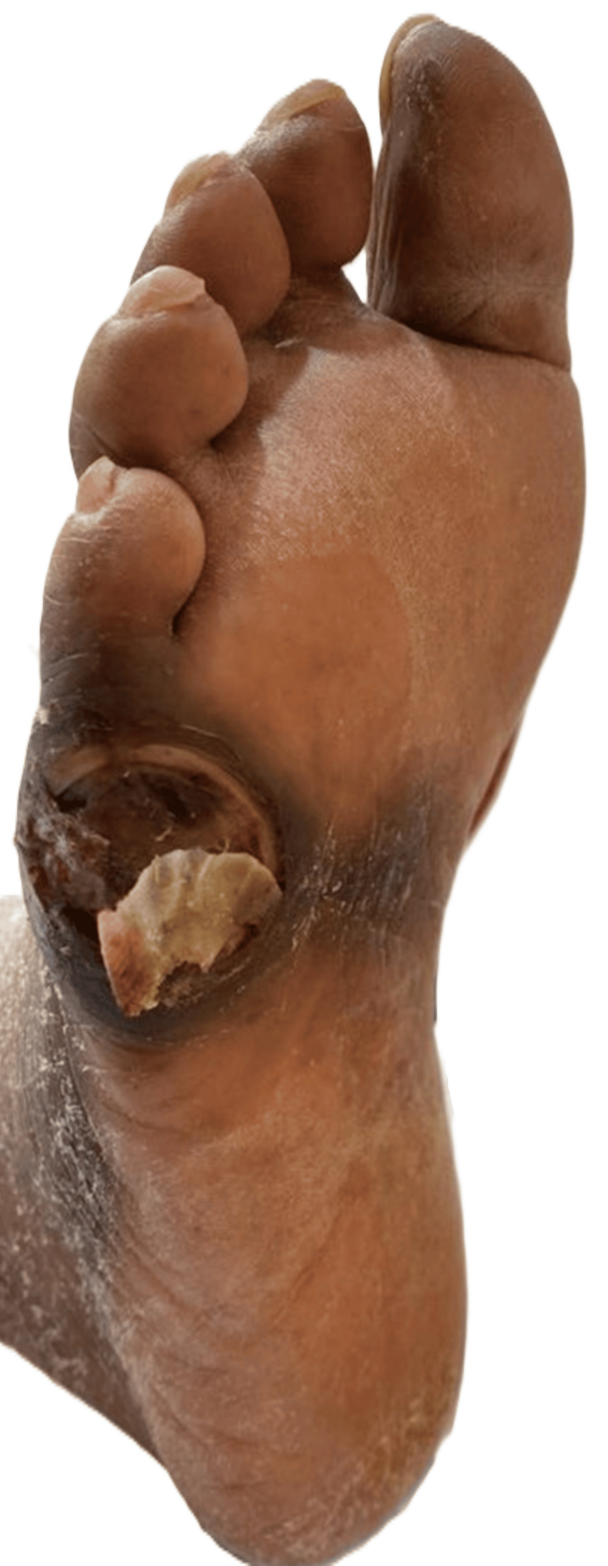
A photograph of the lateroplantar aspect of our patient’s foot demonstrating an open wound at the right fifth metatarsal with associated bone exposure.

On arrival, the patient's vital signs were as follows: temperature 97.9°F (36.6°C), blood pressure 127/78 mmHg, pulse 100 beats per minute, respiratory rate 16 breaths per minute, oxygen saturation (SpO₂) 99% on room air, and body mass index (BMI) 17.23 kg/m². The physical exam was remarkable only for cachexia, global xerosis cutis, surgical below-knee absence of the left lower extremity (surgical site well-healed and completely closed) with prosthesis, and a well-circumscribed, full-thickness ulceration with a mixed fibrogranular base overlying the lateroplantar aspect of the right fifth metatarsal head (Figure 1). While there was no associated tenderness, erythema, swelling, fluctuance, serous/purulent drainage, or crepitus of the wound, there was malodor and locally increased calor. The lack of drainage, erythema, crepitus, bullae, ecchymosis, and tenderness to palpation altogether lowered initial clinical suspicion for necrotizing fasciitis, which commonly occurs secondarily to DFIs.

Testing results

Abnormal results from the basic metabolic panel, magnesium, phosphorus, complete blood count, C-reactive protein (CRP), erythrocyte sedimentation rate (ESR/sed rate), urinalysis with microscopic evaluation, mixed venous blood gas, β-hydroxybutyrate, and Laboratory Risk Indicator for Necrotizing Fasciitis (LRINEC) score on admission are listed below (Table 1) [19]. Blood ethanol was undetectable, nares were PCR-negative for methicillin-resistant *S. aureus* (MRSA), bilateral axilla/groin were polymerase chain reaction (PCR)-negative for *Candida auris*, serum osmolality was normal, and the lipid panel was within normal limits. A complete metabolic panel performed shortly after admission showed normal total bilirubin levels and no elevation in aspartate transaminase (AST), alanine transaminase (ALT), or alkaline phosphatase (ALP) levels.

**Table 1:** Abnormal values from laboratory testing performed on admission NA: sodium; K: potassium; CL: chloride; CA: calcium; MG: magnesium; PHOS: phosphorus; WBC: white blood cell count; HGB: hemoglobin; HCT: hematocrit; MCHC: mean corpuscular hemoglobin concentration; CRP: c-reactive protein; ESR: erythrocyte sedimentation rate; LRINEC:Laboratory Risk Indicator for Necrotizing Fasciitis

Component	Result	Reference Ranges and Units
Na	130	136 – 144 mmol/L
K	3.1	3.3 – 5.1 mmol/L
Cl	88	98 – 108 mmol/L
Anion gap	18	4 – 16
Blood glucose	628	70 – 100 mg/dL
Ca	8.6	8.9 – 10.3 mg/dL
Mg	1.4	1.8 – 2.5 mg/dL
Phos	2.2	2.5 – 4.7 mg/dL
WBC	10.8	3.5 – 10.5 k/uL
Hgb	9.8	13.0 – 17.5 g/dL
HCT	30.9	38.0 – 54.0%
MCHC	31.7	32.0 – 36.0 g/dL
CRP	103.8	< 8.1 mg/L
ESR	> 119	0 – 20 mm/hr
Urine glucose	3+	Negative
Urine ketones	Trace	Negative
Blood lactate	2.7	0.9 – 1.7 mmol/L
β-hydroxybutyrate	2.5	0.0 – 0.3 mmol/L
LRINEC score	5	0 – 5

**Figure 2:**
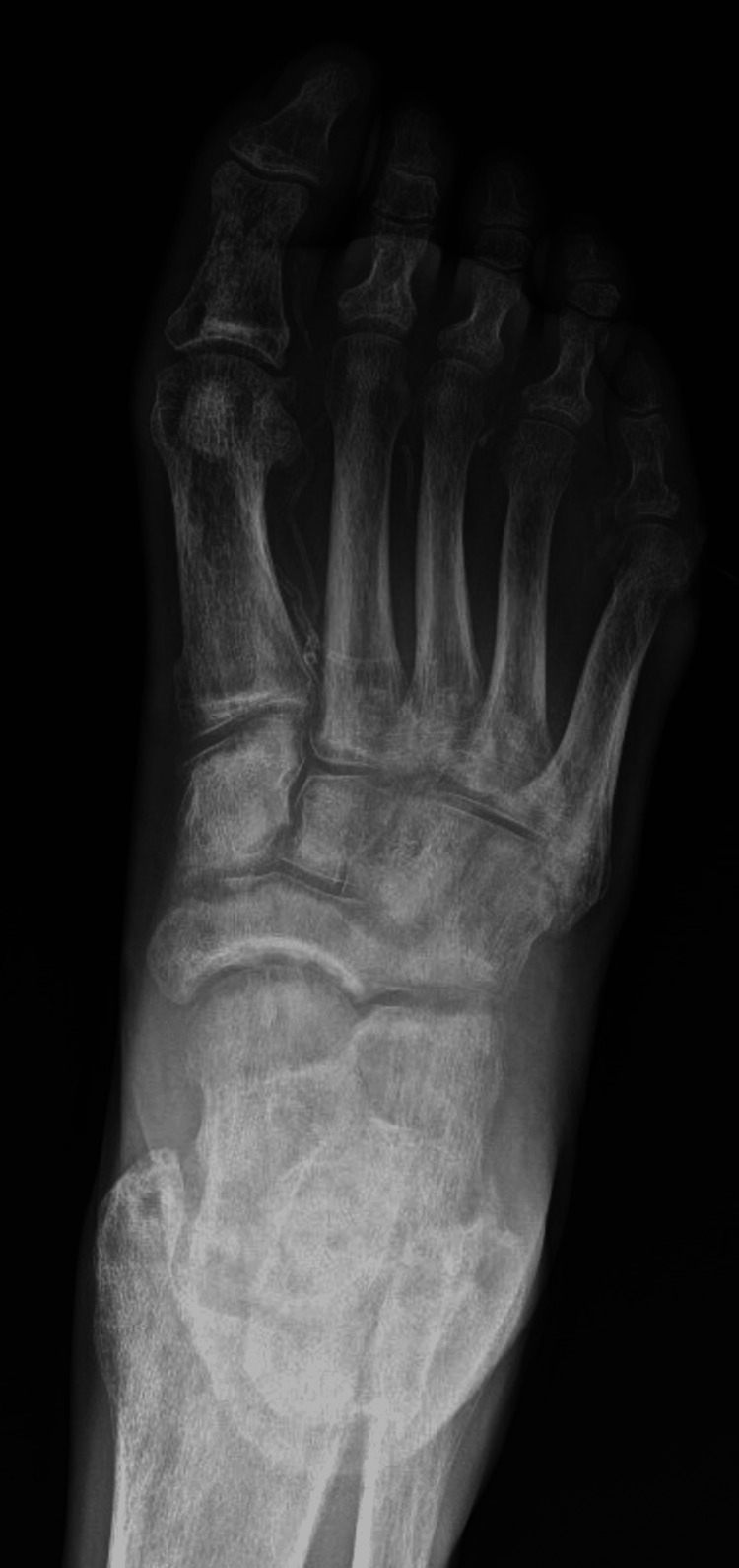
Anterior-posterior radiograph of our patient's right foot

**Figure 3:**
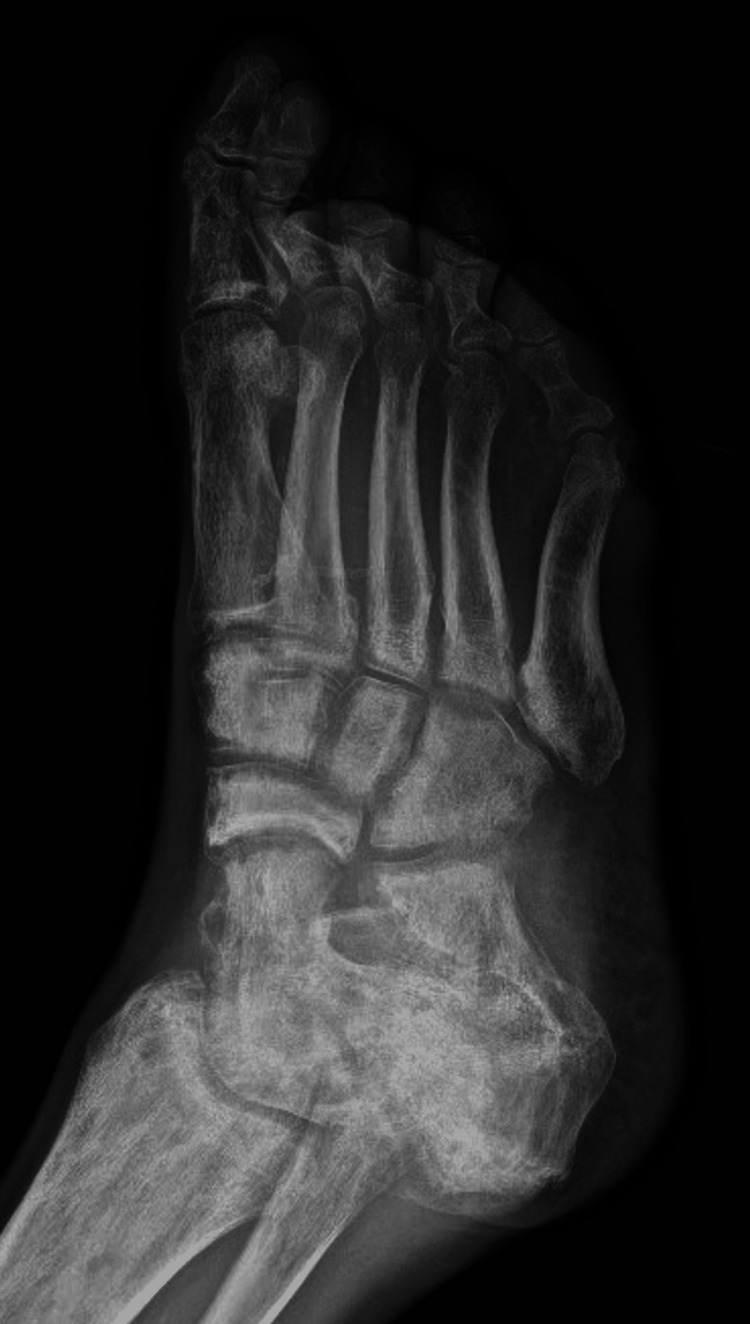
Oblique radiograph of our patient's right foot

Anterior-posterior (Figure 2), oblique (Figure 3), and lateral (not shown) radiographic imaging of the right foot revealed ulceration of the lateral right foot with probable exposure of the fifth metatarsal head without definitive findings of active osteomyelitis.

Non-invasive vascular studies were consistent with mild arterial disease: the right ankle-brachial index was 0.81 - 0.94 (reference range: 1.0 - 1.4), Doppler ultrasonography of the right lower extremity revealed loss of the dicrotic notch in the pulse volume recording waveform, and the right great toe-brachial index was 0.35 (reference: > 0.7) with a toe systolic pressure of 41 mmHg (reference: ≥ 50 mmHg (≥ 65 mmHg has been suggested for patients with DM)) and reduced photoplethysmography waveforms in all five digits [20-22]. However, angiography of the right lower extremity revealed all surveyed arteries to be patent, including the wound-supplying vessel, the lateral plantar artery. Taking into account our patient's clinical picture, vascular study findings were consistent with DM-associated microangiopathy.

Culture from the bone biopsy performed at bedside grew moderate colonies of milleri-group streptococci (*S. anginosus*, *S. constellatus*, and *S. intermedius*) and unidentified anaerobes. Blood cultures showed no growth of bacteria or yeast by day five.

Tissue specimens obtained during the partial fifth ray amputation revealed subepidermal soft tissue with coagulative necrosis and abundant acute inflammation, but viable subadjacent bone with reactive changes, granulation tissue, and associated acute inflammation. Analysis of the bone clearance fragment revealed viable bone with sclerosis, marrow stromal edema, and acute inflammation. There was no evidence of fungi or acid-fast bacilli.

Hospital course

In the ED, our patient was treated for hyperosmolar hyperglycemic state with potassium repletion, dextrose 50% in water (D50W), glucagon, and insulin lispro. Despite aggressive use of insulin (glargine at bedtime, lispro with meals, and lispro after meals as needed), blood glucose levels consistently below 180 mg/dL were difficult to achieve for the course of our patient's admission.

The podiatry team was consulted and obtained a bone biopsy in the ED. Our patient was then admitted to the floor and immediately started on empiric intravenous (IV) piperacillin/tazobactam and IV vancomycin. Our patient developed postprandial diarrhea on day two of admission, and stool PCR was positive for both norovirus genogroup (G) I/GII and toxigenic *C. difficile*, so a standard course of oral vancomycin was added to our patient's antibiotic regimen.

Following culture results, our patient was switched to IV ceftriaxone and IV metronidazole for two days before our consulting podiatry team deemed a partial fifth ray amputation with fillet toe flap was necessary due to the extent of tissue destruction. Intraoperatively, the base of the right fifth proximal phalanx was found to be fragile and grey-colored, findings consistent with osteomyelitis. 

Our patient was switched to oral amoxicillin-clavulanate following amputation, continued for five days in the setting of clear surgical margins, and discharged two days after amputation.

## Discussion

DFIs and associated osteomyelitis, especially in the setting of peripheral neuropathy, are a leading cause of morbidity in patients with DM that frequently necessitate aggressive inpatient intervention [23]. DFIs can rapidly lead to sepsis and become life-threatening, as even well-controlled DM causes immune dysfunction, and because DFIs can go unnoticed amid peripheral neuropathy, a common sequela of DM. As DFIs take a considerable toll on patients’ well-being and contribute to the rising costs of healthcare, it is important they are treated correctly and quickly to prevent deterioration to osteomyelitis and/or sepsis. 

It is unclear why *S. milleri* is so infrequently isolated from DFIs. It may be such that its normal habitat in the GI/GU tracts makes translocation to the feet and subsequent survival difficult. As well, the proclivity of *S. milleri *to form pyogenic/loculated, deep-space abscesses may render it unlikely to survive in the comparatively superficial and polymicrobial environment of a DFI. It is also possible that *S. milleri* is simply underreported; perhaps the rarity of *S. milleri* in osteomyelitis is overlooked since the greater *Streptococcus *genus is so frequently implicated.

It is important to be aware of all microbes implicated in DFIs and osteomyelitis, given the morbidity they inflict on patients and cost to the healthcare system, as well as to increase awareness of the under-recognized behavior of commensal bacteria.

## Conclusions

It is important to recognize milleri-group streptococci as causative organisms in DFIs and osteomyelitis for several reasons. First, *S. milleri* are normal flora of the GI/GU systems, but not skin, and are rarely reported in DFIs/osteomyelitis; as such, isolation of *S. milleri* may reflect extreme immunocompromise or indicate an atypical mechanism of inoculation, such as hematogenous spread/translocation from the GI/GU tract(s). Second, while DFIs typically do not form abscesses, even when having progressed to osteomyelitis, infection with the highly pyogenic *S. milleri *may confer an underappreciated risk of abscess and sinus-tract formation, increasing the risk of morbidity and mortality, especially in patients with DM. And third, being a subtype of viridans-group streptococci,* S. milleri* may have variable susceptibility to empiric antibiotic regimens, and thus its infection may necessitate relatively earlier surgical intervention. 

While it is not clear why *S. milleri* is infrequently reported in DFIs and osteomyelitis, this case supports the notion that these commensal bacteria of the GI/GU tracts may have an enhanced ability to cause foot and associated bone infections in the setting of severely elevated blood glucose levels.
